# Generation and Characterization of a Mouse Model Harboring the Exon-3 Deletion in the Cardiac Ryanodine Receptor

**DOI:** 10.1371/journal.pone.0095615

**Published:** 2014-04-17

**Authors:** Yingjie Liu, Ruiwu Wang, Bo Sun, Tao Mi, Jingqun Zhang, Yongxin Mu, Ju Chen, Michael J. Bround, James D. Johnson, Anne M. Gillis, S. R. Wayne Chen

**Affiliations:** 1 Libin Cardiovascular Institute of Alberta, Departments of Physiology & Pharmacology, and Biochemistry & Molecular Biology, University of Calgary, Calgary, Alberta, Canada; 2 Department of Molecular Biophysics and Physiology, Rush University Medical Center, Chicago, Illinois, United States of America; 3 Department of Medicine, University of California San Diego, La Jolla, California, United States of America; 4 Cardiovascular Research Group, Life Sciences Institute, University of British Columbia, Vancouver, British Columbia, Canada; Loyola University Chicago, United States of America

## Abstract

A large genomic deletion in human cardiac ryanodine receptor (*RYR2*) gene has been detected in a number of unrelated families with various clinical phenotypes, including catecholaminergic polymorphic ventricular tachycardia (CPVT). This genomic deletion results in an in-frame deletion of exon-3 (Ex3-del). To understand the underlying disease mechanism of the RyR2 Ex3-del mutation, we generated a mouse model in which the RyR2 exon-3 sequence plus 15-bp intron sequences flanking exon-3 were deleted. Heterozygous Ex3-del mice (Ex3-del^+/−^) survived, but no homozygous Ex3-del mice were born. Unexpectedly, the Ex3-del^+/−^ mice are not susceptible to CPVT. Ex3-del^+/−^ cardiomyocytes exhibited similar amplitude but altered dynamics of depolarization-induced Ca^2+^ transients compared to wild type (WT) cells. Immunoblotting analysis revealed markedly reduced expression of RyR2 protein in the Ex3-del^+/−^ mutant heart, indicating that Ex3-del has a major impact on RyR2 protein expression in mice. Cardiac specific, conditional knockout of the WT RyR2 allele in Ex3-del^+/−^ mice led to bradycardia and death. Thus, the absence of CPVT and other phenotypes in Ex3-del^+/−^ mice may be attributable to the predominant expression of the WT RyR2 allele as a result of the markedly reduced expression of the Ex3-del mutant allele. The effect of Ex3-del on RyR2 protein expression is discussed in relation to the phenotypic variability in individuals with the RyR2 exon-3 deletion.

## Introduction

The cardiac Ca^2+^ release channel (ryanodine receptor type 2, RyR2) plays an essential role in excitation-contraction (EC) coupling and sarcoplasmic reticulum Ca^2+^ handling in the heart [1]. RyR2 is also a key player in the pathogenesis of cardiac arrhythmias and cardiomyopathies [2]. To date, more than 150 mutations in RyR2 have been associated with different forms of cardiac arrhythmias, including catecholaminergic polymorphic ventricular tachycardia (CPVT), catecholaminergic idiopathic ventricular fibrillation, and atrial fibrillation [2]–[5]. Most patients with RyR2 mutations exhibit structurally normal hearts. However, some RyR2 mutations have been linked to cardiomyopathies as well as cardiac arrhythmias [6]–[11]. For instance, a large genomic deletion (1.1–3.6 kb) in the *RYR2* gene that covers exon-3 was identified in a number of unrelated families [11]–[14]. Individuals with this exon-3 deletion display a wide spectrum of clinical phenotypes, including CPVT, sinoatrial node dysfunction, bradycardia, atrial fibrillation, AV block, dilated cardiomyopathy, and left ventricular non-compaction.

The deletion of exon-3 results in an in-frame deletion of 35 amino acid residues (Asn57-Gly91) in the NH_2_-terminal region of the RyR2 channel that is believed to be important for channel function [15]–[18]. Interestingly, the missing structural element due to exon-3 deletion can be rescued by a β-strand switching [16], [17]. As a result, the exon-3 deletion does not grossly affect the overall folding of the NH_2_-terminal domain of RyR2. Consistent with these observations, functional studies in HEK293 cells revealed that the exon-3 deleted RyR2 mutant remained functional, but displayed altered properties of Ca^2+^ release activation and termination [18].

Although the structural impact of the exon-3 deletion and its effect on spontaneous Ca^2+^ release in the heterologous HEK293 cells have been well characterized, the mechanisms by which the exon-3 deletion in RyR2 causes cardiac arrhythmias and cardiomyopathy are unknown. Genetically engineered mouse models harboring disease-associated mutations have been widely used for studying disease mechanisms. Indeed, a number of knock-in mice expressing RyR2 mutations linked to CPVT have been generated and characterized [19]–[26]. These RyR2 mutant mice have provided important insights into how RyR2 mutations cause CPVT. However, in contrast to CPVT-associated RyR2 mutations, there are few models that express cardiomyopathy-associated RyR2 mutations. In an attempt to understand the disease mechanism underlying RyR2-associated cardiomyopathies, in the present study, we generated a mouse model in which the entire exon-3 and part of the introns 2 and 4 were deleted using the knock-in approach. Unexpectedly, we found that this deletion has a dramatic impact on the expression of the mouse RyR2 protein. The observation that the exon-3 deletion alters the expression of RyR2 may provide some clues to the various clinical phenotypes and variable severities of patients with the RyR2 exon-3 deletion [11]–[14].

## Materials and Methods

### Generation of a mouse model harboring the RyR2 exon-3 deletion

A genomic DNA phage clone containing part of the mouse cardiac ryanodine receptor gene was isolated from the lambda mouse 129-SV/J genomic DNA library (Stratagene) and used to construct the RyR2 exon-3 deletion (Ex3-del) knock-in (KI) targeting vector. This genomic DNA fragment (about 15 kb) was released from the lambda vector by NotI, and subcloned into pBluescript to form the RyR2 genomic DNA plasmid. PCR-based site-directed mutagenesis was performed to generate a 660 bp DNA fragment containing the Ex3-del mutation using this RyR2 genomic DNA plasmid as a template. An XhoI site was created in the 5′ and a BamH I site in the 3′ in this DNA fragment. The modified XhoI-BamHI fragment was then subcloned into the targeting vector that contains a neomycin selection cassette flanked by FRT sites using BamH I and XhoI. The 2186 bp HindIII-HindIII and the 5994 bp AflII-AflII genomic DNA fragments were isolated from the RyR2 genomic DNA plasmid and inserted into the targeting vector to form the 5′ arm via the HindIII sites and the 3′ arm via the AflII sites, respectively. The DNA sequences of all PCR fragments used for constructing the targeting vector were confirmed by DNA sequencing. The targeting vector was linearized with NotI and subsequently electroporated into R1 embryonic stem (ES) cells.

G418-resistant ES clones were screened for homologous recombination by Southern blotting using an external probe. Briefly, genomic DNA was extracted from G418-resistant ES cell clones. ES cell DNA was digested using BglII, separated on a 0.8% (wt/vol) agarose gel, and subsequently blotted onto a nitrocellulose membrane. A DNA probe (∼700 bp) was generated by PCR from mouse genomic DNA using the specific primers, forward: 5′-TGCCTTGTCGTCAATTAAGCTGT-3′; and reverse: 5′-TACATGTGTGCAGTTGCCCATA-3′. The PCR product was subsequently radiolabeled using [^32^P]dCTP by random priming (Invitrogen). DNA blots were hybridized with the radiolabeled probe and visualized by autoradiography. Eight positive homologous recombinants were detected out of 780 ES cell clones, two of which were microinjected into blastocysts from C57BL/6J mice to generate male chimeras. Male chimeras were bred with female 129sve mice to generate germline transmitted heterozygous Ex3-del neo knock-in mice. RyR2 Ex3-del neo male mice were bred with female mice that express Flp recombinase to remove the selectable marker (the neomycin resistant gene, neo). The genotypes from F1 generation without neo were determined by PCR using DNA from tail biopsy specimens using the DNeasy Tissue Kit from Qiagen and the DNA primers, forward: 5′-CACAGACACACAGTAAGGCATTAC-3′; reverse: 5′-GTATGTCTTTCAGACATCCTAAGC -3′.

All animal studies were approved by the Institutional Animal Care and Use Committees at the University of Calgary, and performed in accordance with NIH guidelines. RyR2 WT and mutant mice (1.5–9 months of age) were used for all experiments.

### Confirmation of exon-3 deletion at the mRNA level using RT-PCR

Total RNA was isolated from about 100 mg of heart tissues from RyR2 exon-3 deletion mutant mice or RyR2 wild type mice using the RNA isolation kit (Invitrogen) according to the manufacturer's instructions. First-strand cDNA was synthesized from ∼5 µg of total RNA using Superscript II RNase H reverse transcriptase (Invitrogen) with gene-specific primer (5′-ATAGCCTTGGGCTGCTTCACTTCC) at 50°C. Amplification of cDNA fragments by polymerase chain reaction (PCR) was performed in a 50 µl mixture that contains 1 µl cDNA, 10 µl 5× PCR buffer, 0.5 µM of each forward or reverse gene-specific primer (Forward primer: 5′-TGATGCGGGCGAAGGCGAGGAT; Reverse primer:5′-GAAGGCAGCATCCACATGCCAGCT), 200 µM dNTP, and 0.01 U *Q5* High-Fidelity DNApolymerase (New England Biolab). PCR was performed in a thermocycler with an initial denaturation at 98°C for 30 seconds, followed by 30 cycles (10 s at 98°C, 15 s at 68°C, and 30s at 72°C) of amplification, and a final cycle of 2 min at 72°C. PCR products were separated by 1.8% agarose gel electrophoresis, and desired DNA fragments were purified using QIAGEN DNA Extraction kit and cloned into pBluescript vector. Plasmid DNA was isolated using QIAGEN Plasmid Purification kit. Insert-positive clones were identified by restriction enzyme digestion (EcoRI and HindIII), and their sequences were determined by DNA sequencing.

### Generation of inducible, cardiac specific, conditional RyR2 knockout mice containing RyR2 exon-3 deletion

The conditional RyR2 knockout (KO) mouse model (RyR2-floxed mice) was generated as described previously [27]–[29]. These RyR2-floxed mice were crossed with the myosin heavy chain (MHC)-mer-Cre-mer mice that express the tamoxifen-inducible, MHC promoter-controlled Cre-recombinase [30]. This breeding produced tamoxifen-inducible, cardiac-specific, heterozygous RyR2 conditional KO mice (iRyR2^wt/flox^), which were used to generate tamoxifen-inducible, cardiac-specific, homozygous RyR2 conditional KO mice (iRyR2^flox/flox^). These iRyR2^flox/flox^ mice were used to conditionally and specifically diminish the expression of the WT RyR2 allele. To do this, we bred the iRyR2^flox/flox^ mice with the RyR2 Ex3-del^+/−^ mutant mice to generate the iRyR2^flox/Ex3-del^ mice. For induction of knockout of the WT RyR2 allele, tamoxifen (sigma, 75 mg/kg/day) was injected (intraperitoneal, i.p.) into iRyR2^flox/Ex3-del^ and iRyR2^flox/flox^ mice (1.5–2 months) for 3 consecutive days. The mice were used for stress tests and immunoblotting analysis ∼12 days post tamoxifen treatment.

### ECG recordings

RyR2 Ex3-del^+/−^ mutant mice and their WT littermates, iRyR2^flox/Ex3-del^, and iRyR2^flox/flox^ mice were assessed for their susceptibility to stress-induced ventricular tachyarrhythmias using ECG recording [24]. Briefly, mice were lightly anesthetized with isoflurane vapor (0.5–1%) and 95% O_2_. Anesthetized mice were placed on a heating pad (27°C) and needle electrodes were inserted subcutaneously into the right upper limb and left lower abdomen for ECG recording (BIOPAC MP System, Goleta, CA). The animals′ ECG was continuously monitored under anesthesia until the heart rate became stabilized. Baseline ECG was recorded for 5–10 minutes. For induction of ventricular arrhythmias, the mice were subjected to intraperitoneal injection of a mixture of epinephrine (1.6 mg/kg) and caffeine (120 mg/kg). ECG was continuously recorded for 30 minutes after the injection of epinephrine and caffeine.

### Western blotting

Mouse hearts were crushed by a Wollenberger clamp pre-cooled in liquid nitrogen. The crushed heart tissues were stored at −80°C until use. Frozen heart tissues were pulverized in liquid nitrogen and homogenized immediately in 6 volumes of homogenizing buffer containing 30 mM KH_2_PO_4_ (pH 7.0), 40 mM NaF, 5 mM EDTA, 300 mM sucrose, 4 µM leupeptin, 1 mM benzamidine, 100 µM PMSF, and 0.5 mM DTT, aliquoted, frozen with liquid nitrogen and stored at −80°C until use. Aliquots of homogenates were solubilized in a final 500 µl of solubilizing buffer containing 50 mM Tris-HCl (pH 7.4) plus 3% SDS for 1 h at room temperature and then incubated at 55°C for 10 min. The insoluble materials were removed by centrifugation at 12,000×g for 10 min [31]. The protein concentration of the supernatant was determined using a BioRad detergent-compatible protein assay kit. Solubilized proteins (15–20 µg) were used for SDS-PAGE [32]. RyR2 proteins resolved in 6% SDS-PAGE were transferred to nitrocellulose membranes at 45 V for 18–20 h at 4°C in the presence of 0.01% SDS according to the method of Towbin et al [33]. For the detection of β-actin, proteins were separated in 15% SDS-PAGE and transferred at 110 V for 1 h at 4°C. The nitrocellulose membranes containing the transferred proteins were blocked for 30 min with phosphate buffered saline (PBS) containing 0.5% Tween-20 and 5% skim milk powder. The blocked membrane was incubated with anti-RyR2 or anti-β-actin antibodies, and washed with PBS containing 0.5% Tween-20 for three times with shaking, each time for 5 min. The membrane was then incubated with the secondary anti-mouse or anti-rabbit IgG (H&L) antibodies conjugated to horseradish peroxidase (1∶20,000) for 30 min. After washing with PBS containing 0.5% Tween-20 for three times, the bound antibodies were detected using an enhanced chemiluminescence kit from Pierce. The intensity of each band was determined from its intensity profile obtained by ImageQuant LAS 4000 (GE Healthcare Life Sciences), analyzed by using the Image-J software, and normalized to that of β-actin.

### Single cell Ca^2+^ imaging of isolated ventricular myocytes

Ventricular myocytes were isolated using retrograde aortic perfusion as described previously [34]. Isolated cells were kept at room temperature in Krebs-Ringers-HEPES (KRH) buffer (in mM: 125 NaCl, 12.5 KCl, 25 HEPES, 6 glucose, and 1.2 MgCl_2_, pH 7.4) containing 20 mM taurine, 20 mM 2,3-butanedione monoxime (BDM), 5 mg/ml albumin, and 1 mM free Ca^2+^ until use. Freshly isolated mouse ventricular myocytes were added to glass coverslips pre-coated with 50 µg/ml laminin, and loaded with 5 µM Rhod-2, AM (Molecular Probes, USA) in KRH buffer containing 1 mM Ca^2+^ for 20 min at room temperature as described previously [35]. The glass coverslip pre-mounted to a recording chamber was then placed onto an inverted microscope (Nikon ECLIPSE Ti) equipped with a Nikon CFI Plan Apo VC 60xWI objective. Then Rhod-2 loaded cells were perfused with KRH buffer containing 2 mM extracellular Ca^2+^ and 5 µM blebbistatin. The cells were paced by field stimulation using the S88X electric stimulator (Grass, USA) at 3 Hz. Confocal line-scanning (512 pixels and 1.9 ms per line) were performed along the longitudinal axis of cells for 10 seconds using the Nikon A1R confocal system. The Rhod-2 loaded myocytes were excited using the 561 nm diode laser and the fluorescence emission at 570–620 nm was recorded. The line-scan images were processed and analyzed with the NIS-Elements AR 4.0.

### Statistical analysis

All values shown are mean ± SEM unless indicated otherwise. To test for differences between groups, we used unpaired Student's *t* tests (2-tailed). A *P* value <0.05 was considered to be statistically significant.

## Results

### Generation of a mouse model expressing the RyR2 exon-3 deletion mutant

**Figure 1:**
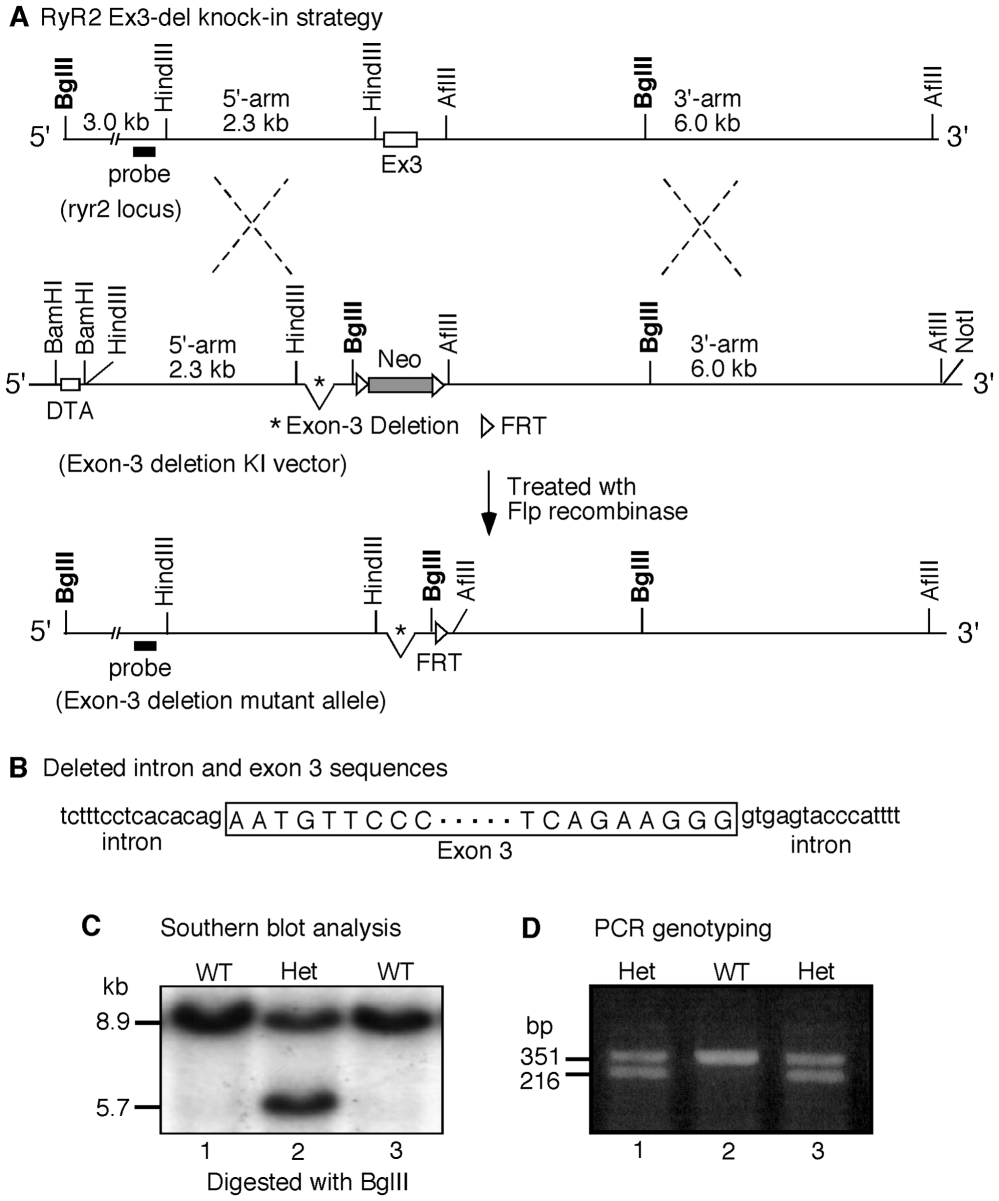
Generation of the RyR2 Ex3-del mutant mouse model. (A) The mouse *ryr2* locus (top line), the construction of the knock-in (KI) vector containing the deletion of the exon-3 and flanking intron sequences in the mouse *ryr2* gene, the selectable markers neo (neomycin resistant gene) and DTA (diphtheria toxin A), the FRT sites (middle line), the generation of the exon-3 deletion mutant allele, and the removal of the neo gene via homologous recombination (bottom line) are depicted. (B) Intron and exon sequences that were deleted in the RyR2 Ex3-del mutant mice. (C) Southern blot analysis of recombinant embryonic stem cells (WT, wild type; Het, heterozygous). (D) PCR genotyping using tail samples from WT and Ex3-del heterozygous (Het) mice.

Naturally occurring deletion of exon-3 (Ex3-del) encoding residues Asn57-Gly91 in the NH_2_-terminal region of the RyR2 channel has been linked to various cardiac abnormalities in humans, but their causal mechanisms are unknown [11]–[14]. To determine the physiological consequence of this deletion, we generated a mouse model harboring the RyR2 Ex3-del mutant. As shown in Fig. 1, the 105-bp exon-3 sequence and the intron sequences (15-bp on both sides) that flank exon-3 were removed from an RyR2 genomic DNA fragment. This modified RyR2 DNA fragment was then used to construct the Ex3-del knock-in plasmid (Fig. 1A,B). Mouse embryonic stem (ES) cells were transfected with the Ex3-del knock-in plasmid. Southern blot screening of transfected ES cells identified 8 positive recombinant ES cell clones (Fig. 1C). One of these positive ES cell clones was then used to produce heterozygous RyR2 Ex3-del mutant mice (Ex3-del^+/−^) (Fig. 1D). Interestingly, while heterozygous Ex3-del mice survived and showed no obvious gross defects, no homozygous RyR2 Ex3-del mice were ever detected, suggesting that homozygous RyR2 exon-3 deletion is embryonically lethal in mice.

**Figure 2:**
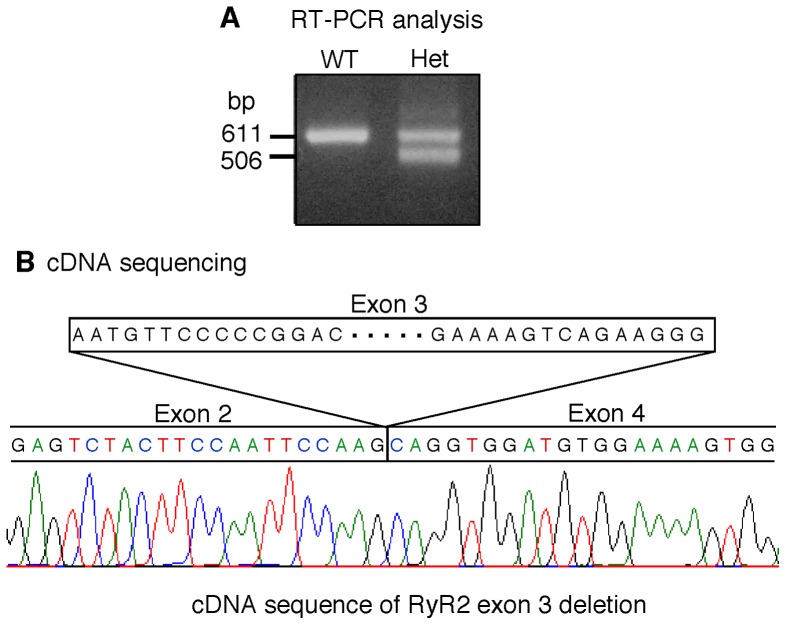
Deletion of exon-3 in the RyR2 mRNA from heterozygous RyR2 Ex3-del mice. A fragment of the mouse RyR2 mRNA covering exon-3 was converted to cDNA and amplified using RT-PCR from total RNAs isolated from wild type (WT) and heterozygous RyR2 Ex3-del (Het) mutant mice (A). The RT-PCR products were isolated and sequenced. The sequence of the RyR2 Ex3-del cDNA was shown (B). Note that the exon-2 sequence is directly followed by the exon-4 sequence, i.e. the exon-3 sequence has been deleted.

To confirm the absence of exon-3 in the Ex3-del^+/−^ mice at the mRNA level, we isolated mRNAs from the wild type (WT) and Ex3-del^+/−^ mouse hearts and generated cDNA fragments covering exon-3 via RT-PCR. Sequencing analysis of these cDNA fragments shows that the RyR2 exon-3 sequence in the Ex3-del^+/−^ mouse heart has indeed been deleted (Fig. 2).

### Heterozygous RyR2 Ex3-del mutant mice show no stress-induced ventricular tachyarrhythmias

**Figure 3:**
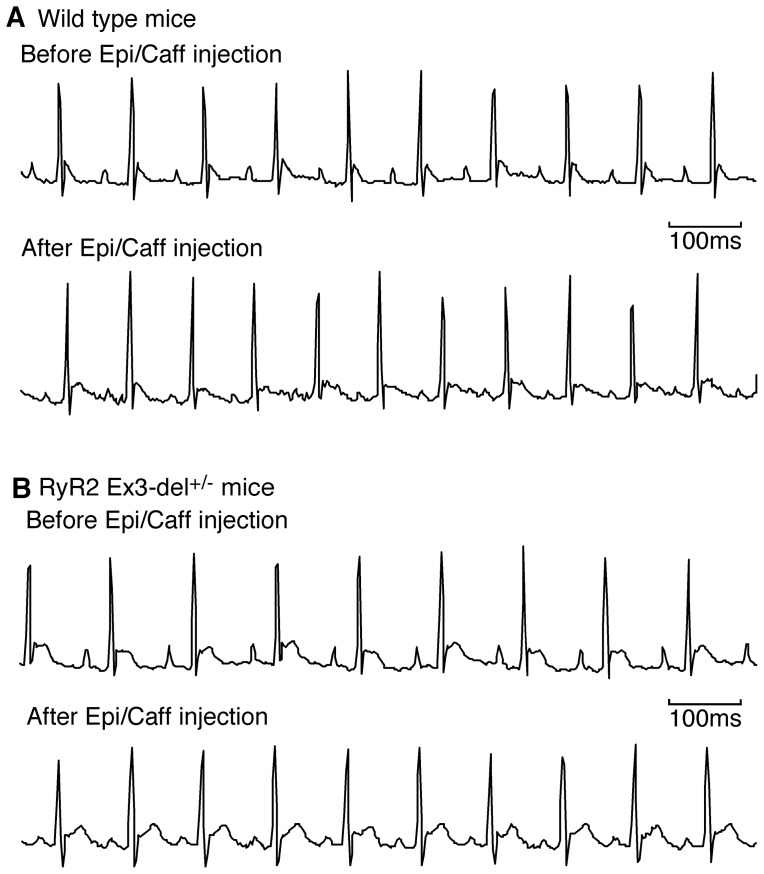
Heterozygous RyR2 Ex3-del mutant mice are not susceptible to CPVT. Representative ECG recordings of wild type (WT) (A) and RyR2 Ex3-del**^+/^** ^−^ mutant (B) mice (1.5–3 months of age) before (top) and after (bottom) the injection of epinephrine (1.6 mg/kg) and caffeine (120 mg/kg). Note that no VTs were detected in either WT (n = 16) or mutant (n = 14) mice during the 30-min period of ECG recording after the injection of the triggers.

Deletion of exon-3 in RyR2 has been associated with CPVT in humans. To assess whether Ex3-del^+/−^ mutant mice are susceptible to stress-induced ventricular tachyarrhythmias (VTs), we monitored the ECG of these mice before and after the injection of pharmacological triggers (caffeine/epinephrine). We have previously shown that these triggers readily induced VTs in the CPVT-linked RyR2 R4496C^+/−^ mutant mice [24]. Unexpectedly, we found that caffeine and epinephrine did not induce VTs in either RyR2 Ex3-del^+/−^ mutant mice (n = 14) or their WT littermates (n = 16) at 1.5–3 months of age (Fig. 3). To assess whether their susceptibility to CPVT is age-dependent, we performed the same caffeine/epinephrine stress tests on mice at 3–5 months of age (5 WT and 10 Ex3-del^+/−^ mice) and 8–9 months of age (8 Ex3-del^+/−^ mice). Similarly, we found that none of these older WT or Ex3-del^+/−^ mutant mice showed stress-induced VTs. Thus, CPVT susceptibility in Ex3-del^+/−^ mutant mice is unlikely to be age-dependent.

### Depolarization-induced Ca^2+^ transients in WT and heterozygous Ex3-del mutant cardiomyocytes

**Figure 4:**
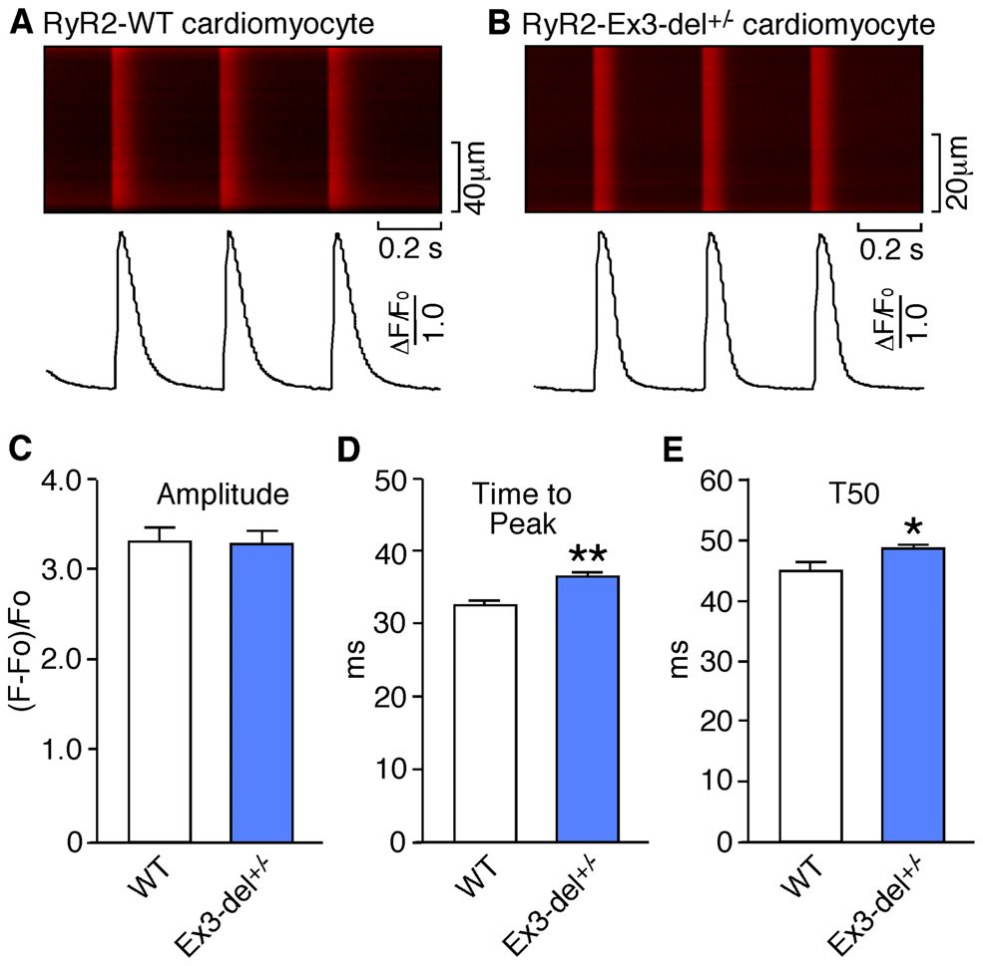
Depolarization-induced Ca^2+^ transients in WT and heterozygous RyR2 Ex3-del mutant cardiomyocytes. Ventricular myocytes isolated from RyR2 WT and Ex3-del**^+/^** ^−^ mutant hearts were loaded with Rhod-2-AM and perfused with 2 mM extracellular Ca^2+^ in KRH solution and paced at 3Hz. Ca^2+^ transients were monitored by line-scan confocal Ca^2+^ imaging. Representative images/traces of WT (A) and Ex3-del**^+/^** ^−^ mutant (B) cardiomyocytes, and average data of the amplitude (C), time to peak (D), and time to 50% decay (E) of Ca^2+^ transients in WT and Ex3-del**^+/^** ^−^ mutant cells are shown. Data shown are mean ± SEM from 35 WT and 58 mutant cells (**P<0.001; *P<0.05).

To determine whether Ex3-del^+/−^ mutant cardiomyocytes exhibit altered excitation-contraction coupling, we analyzed the amplitude and dynamics of Ca^2+^ release evoked by depolarization in cardiac myocytes isolated from RyR2 WT and Ex3-del^+/−^ mutant mice using confocal line-scan Ca^2+^ imaging. As shown in Fig. 4, there was no significant difference (P = 0.472) in the Ca^2+^ transient amplitude (ΔF/F0; WT: 3.32±0.17 vs. Ex3-del^+/−^: 3.29±0.14) between RyR2 WT (n = 35) and Ex3-del^+/−^ mutant (n = 58) ventricular myocytes. On the other hand, the time-to-peak (WT: 32.5±0.7 ms vs. Ex3-del^+/−^: 36.4±0.5 ms, P<0.001) and time-to-50% decay (T50; WT: 44.8±1.5 ms vs. Ex3-del^+/−^: 48.3±0.9 ms, P<0.05) in Ex3-del^+/−^ mutant cells were slightly but significantly increased compared to those in the RyR2 WT cells.

### Heterozygous RyR2 Ex3-del mutant hearts display markedly reduced RyR2 protein expression

**Figure 5:**
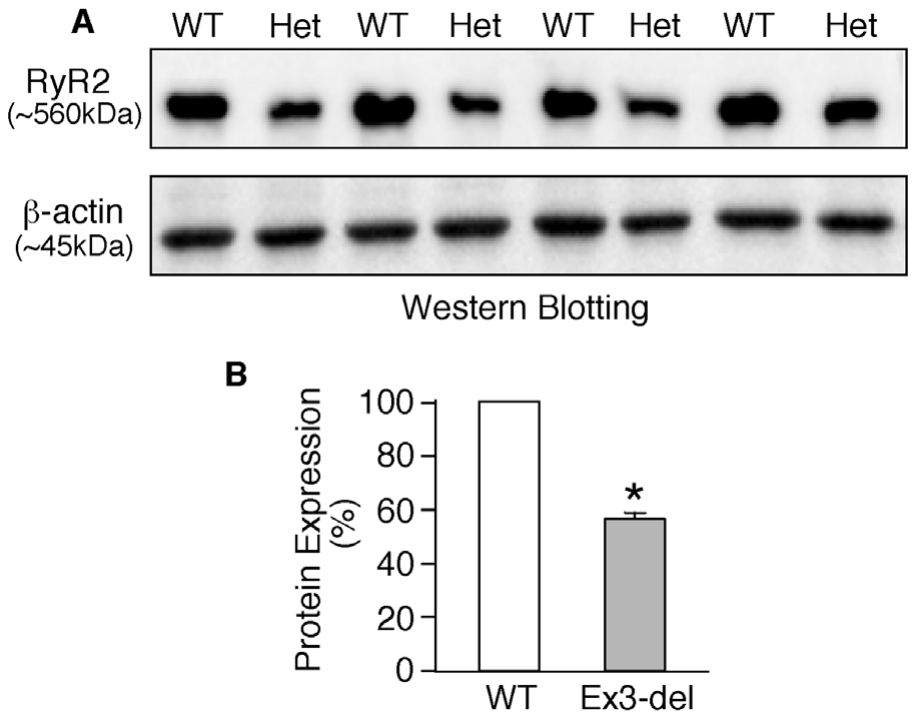
Reduced RyR2 protein expression in heterozygous RyR2 Ex3-del mutant hearts. (A) Whole heart homogenates were prepared from wild type (WT) (n = 4) and RyR2 Ex3-del^−/−^ mutant (n = 4) mice (2–3 months) and used for immunoblotting analysis using antibodies against RyR2 or β-actin. (B) The expression of RyR2 in the Ex3-del hearts was significantly reduced (58±3%) as compared to that in WT hearts (*P<0.001).

The lack of CPVT phenotype in RyR2 Ex3-del^+/−^ mutant mice is surprising given the impact of Ex3-del on RyR2 function in heterologous systems and its link to CPVT and other cardiac abnormalities in patients [11]–[14], [18]. This lack of phenotypes could result from poor expression of the RyR2 Ex3-del mutant protein. To test this possibility, we performed immunoblotting analysis to assess the level of the RyR2 protein expressed in Ex3-del^+/−^ mutant and WT hearts. It should be noted that it is difficult to distinguish the RyR2 Ex3-del mutant protein from the RyR2 WT, as there are currently no specific probes available for the detection of the RyR2 Ex3-del mutant protein. Thus, we examined the total RyR2 protein expressed in the WT and mutant hearts. As shown in Fig. 5, heterozygous Ex3-del mutant hearts (n = 4) exhibited a markedly reduced level of RyR2 protein (58±3%) as compared to WT hearts (n = 4) (P<0.001). Thus, these data indicate that deletion of exon-3 significantly reduces the expression of RyR2 protein in mice. Since the expression of the normal WT RyR2 allele in the heterozygous Ex3-del mutant heart is unlikely to be markedly altered, the reduction in RyR2 expression observed in the heterozygous Ex3-del mutant heart would mostly result from the impaired expression of the Ex3-del mutant allele. Hence, the absence of CPVT phenotype in RyR2 Ex3-del^+/−^ mutant mice may be attributable to the lack of expression of the Ex3-del mutant RyR2 allele.

### Cardiac-specific, conditional knockout of the WT RyR2 allele in heterozygous RyR2 Ex3-del mutant mice

**Figure 6:**
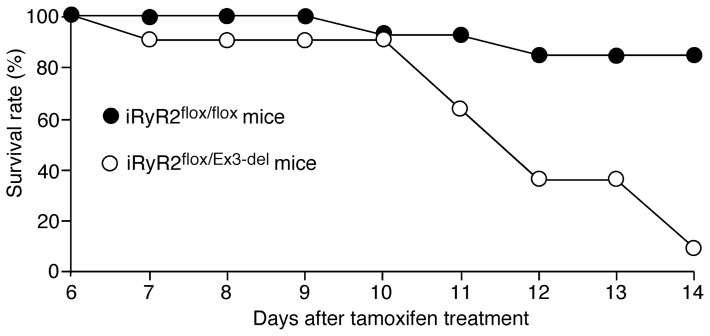
Cardiac-specific, conditional knockout of the WT RyR2 allele in heterozygous RyR2 Ex3-del mutant mice results in early death. iRyR2^flox/flox^ (n = 13, black circles) and iRyR2^flox/Ex3-del^ (n = 11, white circles) mice were injected with tamoxifen (75 mg/kg/day) for 3 consecutive days. The percentage of live mice (survival rate) on day 6–14 post tamoxifen treatment is shown.

To test the possibility that the predominant expression of the WT allele over the mutant allele in Ex3-del^+/−^ mutant mice may mask the impact of the RyR2 Ex3-del mutation, we employed the Cre-Lox recombination technology to conditionally and specifically reduce the expression level of the WT RyR2 allele in the Ex3-del^+/−^ mutant heart. We reasoned that by specifically reducing the expression of the WT allele, we might be able to reveal the impact of the Ex3-del mutation. To this end, we bred the RyR2-floxed mice with the myosin heavy chain (MHC)-mer-Cre-mer mice that express the tamoxifen-inducible, MHC promoter-controlled Cre-recombinase [28]–[30]. Through this breeding, we obtained tamoxifen-inducible, cardiac-specific RyR2 conditional KO mice (iRyR2^flox/flox^) [28], [29]. We then bred these iRyR2^flox/flox^ mice with our RyR2 Ex3-del^+/−^ mutant mice to generate the iRyR2^flox/Ex3-del^ mice. To induce knockout of the WT RyR2 allele, we injected the iRyR2^flox/Ex3-del^ (n = 11) and iRyR2^flox/flox^ (n = 13) mice with tamoxifen. We found that the iRyR2^flox/Ex3-del^ mice died starting ∼11 days post tamoxifen treatment. By 14 days post tamoxifen treatment, most of the iRyR2^flox/Ex3-del^ mice (10 out 11) had died. On the other hand, only 2 of the 13 iRyR2^flox/flox^ mice died 14 days post tamoxifen treatment (Fig. 6). Thus, upon reducing the expression of the WT RyR2 allele, heterozygous Ex3-del mutant mice displayed a higher rate of death than the RyR2-floxed mice.

**Figure 7:**
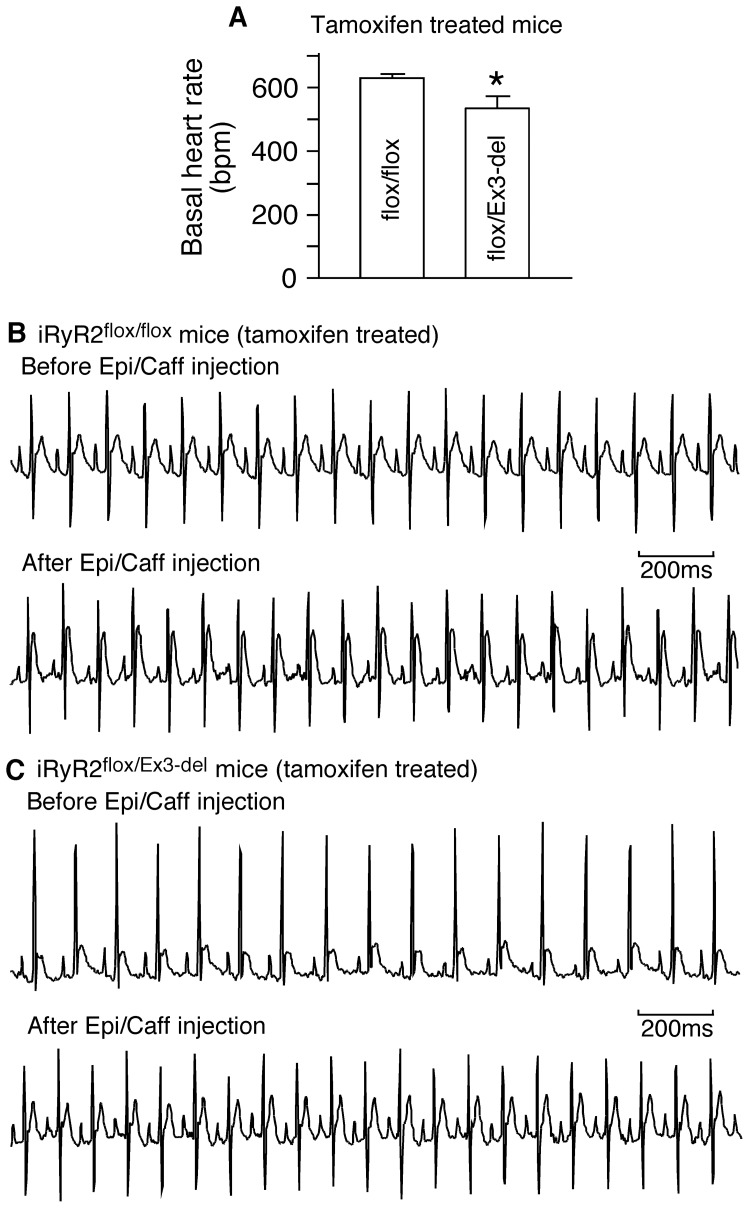
Heterozygous RyR2 Ex3-del mutant mice with cardiac specific, conditional KO of the WT RyR2 allele exhibit bradycardia, but no CPVT. iRyR2^flox/flox^ (n = 11) and iRyR2^flox/Ex3-del^ (n = 8) mice were treated with tamoxifen. ECG recording was performed 12 days post tamoxifen treatment to determine their basal heart rates (before epinephrine/caffeine challenge) (A) and their susceptibility to CPVT (B, C). Representative ECG recordings of the tamoxifen-treated iRyR2^flox/flox^ mice (B) and the tamoxifen-treated iRyR2^flox/Ex3-del^ mice (C) before (top panel) and after (bottom panel) the injection of epinephrine (1.6 mg/kg) and caffeine (120 mg/kg). Note that no VTs were detected in either the tamoxifen-treated iRyR2^flox/flox^ mice (B) or the tamoxifen-treated iRyR2^flox/Ex3-del^ mice during the 30-min period of ECG recording after the injection of the triggers (*P<0.05).

To gain some insight into the cause of death, we monitored the ECG of iRyR2^flox/Ex3-del^ and iRyR2^flox/flox^ mice ∼12 days post tamoxifen treatment. We found that the basal heart rate of the iRyR2^flox/Ex3-del^ mice (535±38 bpm) (n = 8) was significantly reduced as compared to that of iRyR2^flox/flox^ mice (629±13 bpm) (n = 11) (P = 0.012). We also performed caffeine/epinephrine stress tests on these iRyR2^flox/Ex3-del^ and iRyR2^flox/flox^ mice. We found that caffeine/epinephrine challenge did not induce VTs in either the iRyR2^flox/Ex3-del^ (n = 8) or iRyR2^flox/flox^ (n = 11) mice ∼12 days post-tamoxifen treatment (Fig. 7). Thus, upon reducing the expression of the WT RyR2 allele, heterozygous Ex3-del mutant mice displayed bradycardia but no stress-induced VTs.

**Figure 8:**
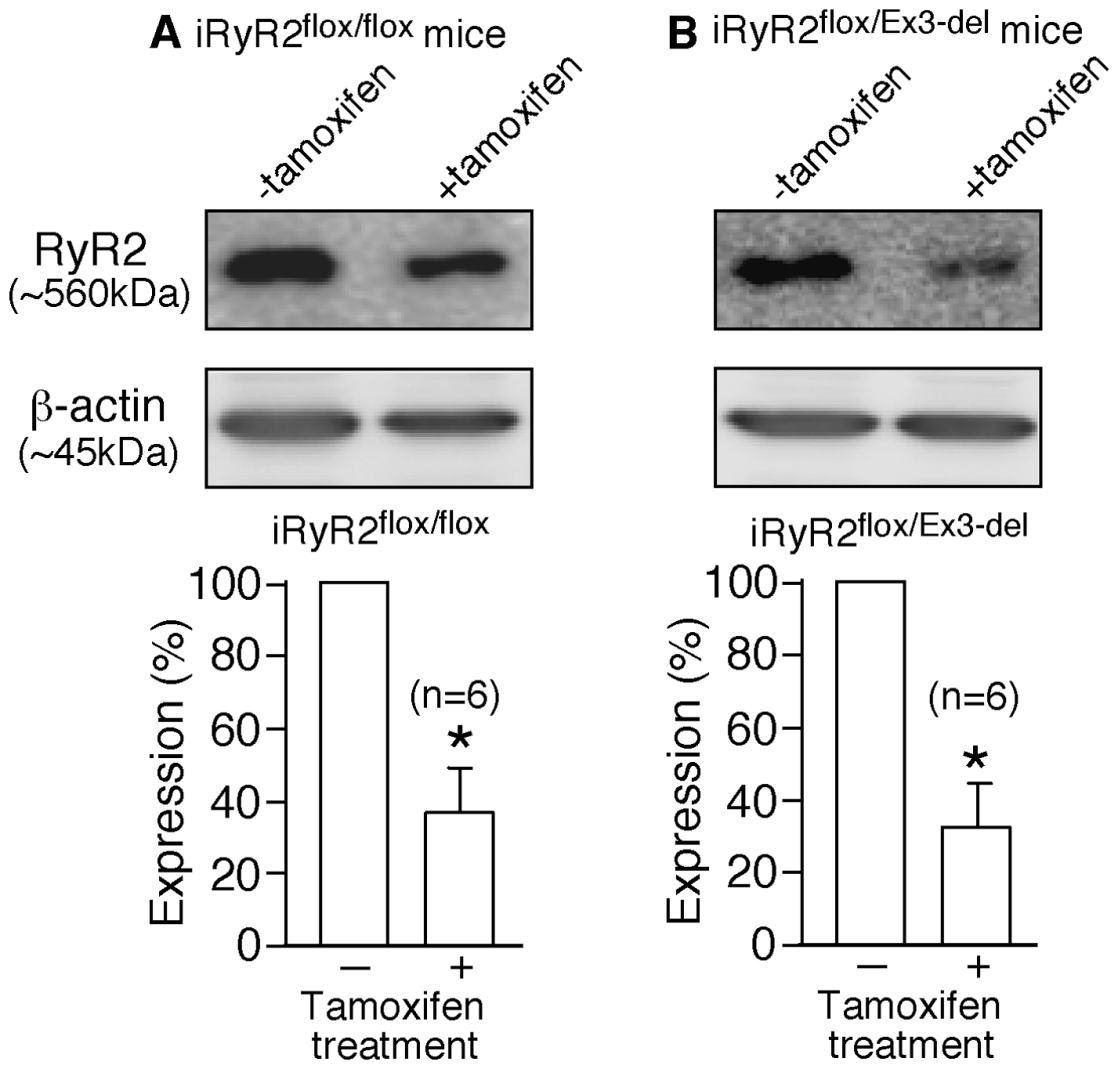
Cardiac specific, conditional knockout of the WT RyR2 allele results in markedly reduced RyR2 expression. Whole heart homogenates were prepared from iRyR2^flox/flox^ (A) and iRyR2^flox/Ex3-del^ (B) mice before and after tamoxifen treatment, and used for immunoblotting analysis using antibodies against RyR2 or β-actin. Note that the expression of RyR2 in iRyR2^flox/flox^ hearts (n = 6) or iRyR2^flox/Ex3-del^ hearts (n = 6) was significantly reduced 12 days post tamoxifen treatment (*P<0.001).

To confirm that tamoxifen treatment did reduce RyR2 expression, we carried out immunoblotting analysis of whole heart homogenates from iRyR2^flox/flox^ and iRyR2^flox/Ex3-del^ mice before and after tamoxifen treatment. Indeed, we found that the expression level of RyR2 in iRyR2^flox/flox^ mouse hearts was significantly reduced to 37±12% of (n = 6, P<0.001) ∼12 days post tamoxifen treatment (as compared to the level before treatment) (Fig. 8). Similarly, the expression level of RyR2 in iRyR2^flox/Ex3-del^ mouse hearts was decreased to 33±8% (n = 6, P<0.001) ∼12 days post tamoxifen treatment (as compared to the level before treatment) (Fig. 8). Taken together, these observations support the notion that the absence of CPVT phenotype in the RyR2 Ex3-del^+/−^ mutant mice is most likely due to the predominant expression of the WT allele as a result of the markedly reduced expression of the Ex3-del mutant RyR2 allele.

## Discussion

Of more than 150 disease-associated RyR2 mutations, exon-3 deletion (Ex3-del) is the only large genomic deletion found in the human *RYR2* gene to date [2], [11]–[14]. Analysis of the genomic DNA sequence reveals that exon-3 in human *RYR2* is flanked by two *Alu* elements. Recombination between these two *Alu* elements due to DNA polymerase slippage during chromosomal replication results in a deletion of 1.1 kb genomic DNA sequence encompassing exon-3 [11]. Similar genomic deletions around exon-3 have been detected in a number of unrelated families, suggesting that the area around exon-3 of the human *RYR2* gene is highly susceptible to *Alu*-mediated genomic rearrangements [11]–[14]. These findings also suggest that the deletion of exon-3 in human RyR2 may represent a common genetic defect and a significant cause of RyR2-associated cardiac abnormalities.

Given its relative high prevalence and potential importance in the pathogenesis of cardiac arrhythmias and cardiomyopathies, we have attempted to generate a mouse model for the human RyR2 exon-3 deletion. To this end, we produced a mouse line in which the entire exon-3 in mouse RyR2 plus a 15-bp intron sequence on both sides of exon-3 were deleted using the knock-in approach. To our surprise and unlike that seen in patients with RyR2 exon-3 deletion, heterozygous Ex3-del mutant mice are not susceptible to stress-induced cardiac arrhythmias. No homozygous Ex3-del mice were born. Immunoblotting analysis revealed that the expression level of RyR2 protein in heterozygous Ex3-del mouse hearts was reduced to 58% of that in WT hearts. This level (58%) represents the total RyR2 protein expression (i.e. WT and mutant proteins together). The dramatic reduction in RyR2 protein expression observed in heterozygous Ex3-del mutant hearts likely resulted from impaired expression of the Ex3-del mutant RyR2 allele as a consequence of the exon-3 deletion. In line with this view, we found that conditionally and specifically knocking-out only the WT RyR2 allele in the heterozygous Ex3-del mutant heart through the Cre-Lox recombination approach resulted in a dramatically reduced level of RyR2 expression. These observations indicate that a large portion of the RyR2 protein expressed in heterozygous Ex3-del mutant hearts is contributed by the expression of the WT allele. In other words, only a small amount of Ex3-del mutant protein is expressed in heterozygous Ex3-del mutant hearts. Based on these observations, we propose that the lack of CPVT and other phenotypes in heterozygous RyR2 Ex3-del mutant mice is likely due to the predominant expression of the WT RyR2 allele because of a dramatically reduced expression level of the Ex3-del mutant protein. In this regard, the expression of RyR2 in heterozygous Ex3-del mutant mice may resemble that in heterozygous RyR2 KO mice. It is important to know that there are no obvious structural and functional abnormalities detected in heterozygous RyR2 KO mice, while homozygous RyR2 KO is embryonically lethal [36]. Similarly, homozygous RyR2 exon-3 deletion is also embryonically lethal, most likely due to the markedly reduced expression level of the Ex3-del mutant protein and the mutation itself.

Individuals heterozygous for the RyR2 exon-3 deletion display a number of clinical phenotypes. These include sinoatrial node dysfunction, atrial arrhythmias, AV block, atrial standstill, bradycardia, dilated cardiomyopathy, or left ventricular non-compaction in addition to CPVT. However, none of these phenotypes were observed in heterozygous RyR2 Ex3-del mutant mice. The predominant expression of the WT RyR2 allele over the Ex3-del mutant allele may mask the impact of the mutant. In support of this view, we found that upon specifically reducing the expression of the WT RyR2 allele, heterozygous Ex3-del mutant mice exhibited bradycardia and death. The exact cause of bradycardia and death in iRyR2^flox/Ex3-del^ mice after tamoxifen treatment is, however, unclear. Bround *et al.* [28] have recently shown that tamoxifen-induced, cardiac specific ablation of RyR2 is sufficient to cause bradycardia and death in mice. Since Ex3-del markedly reduces the protein expression of RyR2, removal of the WT RyR2 allele in iRyR2^flox/Ex3-del^ mice after tamoxifen treatment would further decrease the already reduced level of RyR2 protein expression in iRyR2^flox/Ex3-del^ mice. Such a profound reduction in RyR2 protein expression itself would lead to bradycardia and death [28]. Because of the markedly reduced expression of the Ex3-del mutant protein, the functional impact of Ex3-del on the RyR2 channel is unlikely to contribute significantly to bradycardia and death observed in iRyR2^flox/Ex3-del^ mice. Further investigations are clearly needed to understand how deletion of exon-3 in RyR2 causes bradycardia and sudden death in humans.

The exact mechanism by which deletion of exon-3 affects the expression of the RyR2 protein has yet to be determined. Although deletion of exon-3 is rescued by β-strand switching in the context of the NH_2_-terminal domains of RyR2, it is unclear whether exon-3 deletion affects the stability and turnover of the full-length RyR2 protein in vivo. Furthermore, there may be species differences. The impact of exon-3 deletion on the synthesis and/or degradation of the RyR2 protein in mice and humans may be different. Further studies will be needed to understand how exon-3 deletion leads to a marked reduction in RyR2 expression.

An interesting and important question is whether the deletion of exon-3 affects the expression of the human RyR2 protein in patients. Among the mutant carriers, the clinical phenotypes and severities of patients with the RyR2 exon-3 deletion appear to be variable. For instance, some patients exhibit dilated cardiomyopathies, while others show no consistent structural abnormalities. Further, some patients were characterized with severe sinus bradycardia and atrioventricular block, atrial fibrillation, while others display only bradycardia [11]–[14]. It is possible that variable levels of expression of the RyR2 Ex3-del mutant allele may be attributable, in part, to the various clinical phenotypes and variable severities observed in patients with the RyR2 exon-3 deletion. Analysis of the expression level of the RyR2 protein in heart samples of patients with Ex3-del will be required to test this possibility.

A large number of RyR2 mutations have been associated with cardiac arrhythmias and cardiomyopathies [3]–[5]. The impact of these mutations on the expression of the RyR2 protein is largely unknown. Our present study demonstrates that RyR2 mutations, especially those large genomic rearrangements, may have significant impact on protein expression, thus affecting the ramification of the disease mutations.
